# Unveiling the unknown: first comprehensive assessment of the knowledge, attitudes and practices of hospital cleaning services staff regarding COVID-19 in Lebanon during the pandemic

**DOI:** 10.1186/s13690-023-01149-5

**Published:** 2023-07-17

**Authors:** Dalal Youssef, Linda Abou-Abass, Hamad Hassan

**Affiliations:** 1 Clinical Trial Program, Ministry of Public Health, Beirut, Lebanon; 2 Bordeaux Research Center for Population Health, Institut de sante publique, d’epidemiologie et de developpement (ISPED), Bordeaux University, Bordeaux, France; 3 Lebanese Higher Institute of Technical and Professional, Ministry of Education, Beirut, Lebanon; 4 Neuroscience Research Center, Faculty of Medical Sciences, Lebanese University, Beirut, Lebanon; 5 Ministry of Public Health, Beirut, Lebanon

**Keywords:** Knowledge, Attitudes, Practices, Environmental cleaning staff, Lebanon, COVID-19

## Abstract

**Background:**

Hospital cleaners are the unsung heroes in the fight against the COVID-19 pandemic. This study aimed to assess the knowledge, attitudes, and practices (KAP) of hospital cleaners towards COVID-19 and determine factors associated with good practices.

**Methods:**

A cross-sectional study was conducted in Lebanon between the 1st and 14th November 2020. Using a snowball sampling technique, data were collected through an online survey that was sent to governmental and private hospitals. The questionnaire consisted of socio-demographic characteristics and KAP of hospital cleaners towards COVID-19. Descriptive statistics and logistic regression analysis were performed.

**Results:**

A total of 453 cleaners completed the survey, of whom 54.3% were females. Most participants had a good level of COVID-19 knowledge (98%) and good preventive practices (89.7%). Regarding attitude, 90.7% had a positive attitude toward health facilities, 78.8% toward cleaning and disinfection, and 73.5% toward health authorities. Sociodemographic characteristics, including younger age, higher levels of education, working in private hospitals, and having more than 3 years of experience, were positively associated with good preventive practices. Our results also showed that participants who had good knowledge about COVID-19, COVID-19 prevention and treatment, cleaning and disinfection processes, and COVID-19 risk factors had a higher likelihood of positive preventive practices. Finally, a positive attitude toward health facilities, health authorities, and cleaning and disinfection was positively associated with good practices.

**Conclusion:**

The surveyed cleaners have a high level of knowledge and expressed positive attitudes toward health facilities and health authorities, as well as good preventive practices. Understanding the determinants of cleaning performance is critical in tailoring interventions to improve hospital cleaning.

**Supplementary Information:**

The online version contains supplementary material available at 10.1186/s13690-023-01149-5.

**Table:** 

Text Box 1. Contributions to the literature
• This study comprehensively assesses the KAP of hospital cleaners in Lebanon regarding COVID-19. It highlights the challenges and opportunities specific to their cultural and healthcare context, contributing to the global understanding of cleaners' experiences during the pandemic
• The study addresses the knowledge gaps of cleaners regarding COVID-19 in healthcare settings
• The study explores the attitudes of cleaners towards health facilities. This is crucial for enhancing the work environment and cleaning practices in healthcare settings
• The study’s findings have implications for improving the training provided to cleaners, as well as for enhancing cleaning practices and development of effective strategies to mitigate the spread of COVID-19 in healthcare environments

## Background

The rapid global spread of coronavirus disease 2019 (COVID-19), along with the associated mortality and morbidity, is a cause for alarm [1]. While person-to-person transmission through close contact is the primary mode of COVID-19 transmission, contaminated surfaces can also serve as a source of infection [2, 3]. Generally, surfaces become contaminated when virus-containing droplets land on them or when someone with contaminated hands touches these surfaces. Recent studies indicate that COVID-19 can survive on various surfaces for different durations, ranging from eight hours to several days. For example, it can persist for up to 3 days on plastic, 2–3 days on stainless steel, and 24 h on cardboard [4]. Therefore, it is crucial to decontaminate healthcare environments to reduce indirect transmission of COVID-19 and ensure that healthcare workers (HCWs) and patients have clean and safe settings for work and medical care [5].

Cleaning, disinfection, and proper waste disposal are integral components of the decontamination process. Cleaning refers to the elimination of contaminants, while disinfection involves reducing the presence of persistent microorganisms to a level where they are no longer harmful [6]. Within the realm of enhanced hygiene, hospital environmental service workers (ESWs) are the driving force, playing a crucial role in breaking the chain of infection [7]. Among these essential workers, cleaning staff, in particular, have emerged as unsung heroes in the battle against the COVID-19 pandemic.

The pandemic has had devastating consequences for various occupations, with housekeepers and cleaning staff being severely affected. They are responsible for meticulously cleaning and disinfecting surfaces that could harbor potentially harmful microorganisms. However, hospital cleaning comes with inherent risks and numerous challenges. Cleaners assigned to coronavirus isolation rooms and intensive care units face a constant array of infection hazards as they strive to maintain the proper functioning of hospital spaces. Moreover, they bear the responsibility of preventing the spread of infections within hospital settings. Unfortunately, their behind-the-scenes role, coupled with their status as low-skilled workers, has kept them hidden from the public eye. While healthcare professionals have rightfully received praise worldwide for their life-saving efforts during the pandemic, cleaning staff are often overlooked and underappreciated.

Given the occupational risks faced by cleaning personnel, including exposure to COVID-19, it is crucial to enhance their knowledge and regularly monitor their practices to minimize the risk of infection. Understanding and highlighting their practices can provide valuable insights for improving environmental services measures [8].

In Lebanon, there is a lack of data on the number of COVID-19 infections specifically among cleaning staff in hospitals. However, according to data from the epidemiological surveillance unit, no deaths have been reported in this category. While previous studies conducted in Lebanon have assessed the knowledge and practices of various healthcare professionals, such as physicians [9], dentists [10], pharmacists [11], and nurses [12], no studies have focused on the practices of hospital services staff during the COVID-19 era.

Considering the ongoing COVID-19 pandemic, conducting this study to evaluate the level of knowledge, attitudes, and practices among cleaning services staff working in Lebanese hospitals is of significant importance. Additionally, identifying factors associated with good practices will provide valuable insights for improving overall practices in this context.

## Methodology

### Study tool and design

Due to the Lebanese government's recommendation to minimize in-person interactions during the pandemic, conducting a field-based survey in hospitals was not feasible. Therefore, potential participants were electronically invited to take part in this cross-sectional study. An online survey was conducted during a period of increasing COVID-19 cases in Lebanon, specifically between 1st and 14th November 2020.

### Questionnaire development

A comprehensive literature review was conducted to gather available resources on knowledge, attitudes, and practices (KAP) towards COVID-19. Existing questionnaires on COVID-19 prevention were also examined to identify relevant items and scales, which were incorporated into different sections of the questionnaire. The knowledge section of the questionnaire was developed based on the etiology, transmission, risk, prevention of COVID-19, and recommended cleaning and disinfection procedures [13, 14].

For the attitude section, the theoretical framework of the Health Belief Model (HBM) [15] was utilized and adapted to the context of environmental services. The practice section was based on international guidelines for cleaning and disinfection in healthcare facilities, including recommendations from the World Health Organization (WHO) and the Centers for Disease Control and Prevention (CDC) [14, 16].

To ensure the content validity of the questionnaire, a panel of experts was assembled, consisting of an epidemiologist, a microbiologist, a hygienist, and a biostatistician. The experts evaluated the clarity, relevance, accuracy, and interpretability of the questions in each domain (knowledge, attitude, and practice), selecting the best items to be included.

The questionnaire was initially developed in English and then translated into colloquial Arabic, the official national language of Lebanon, to facilitate administration to local participants. The translation process followed standard guidelines for backward and forward translation, with two forward translations by a sworn translator and an epidemiologist, followed by backward translation by a language communication expert and another epidemiologist to ensure accuracy [17].

Face validity of the questionnaire was assessed by testing it on 10 cleaners and housekeepers. Their feedback on the understanding of the questionnaire items, the significance of the questions, survey flow, readability, layout, style, and absence of confusing wording was carefully evaluated. Based on this face validation process, a revised and finalized version of the questionnaire was produced, incorporating minor modifications and clarifications. This version will be used for the data collection process.

The questionnaire is designed as an anonymous, self-administered tool comprising five sections: (1) socio-demographic characteristics, (2) clinical information, (3) knowledge of cleaning and disinfecting, (4) attitude towards COVID-19, and (5) prevention practices towards COVID-19 among hospital cleaning services staff.

#### Socio-demographic characteristics

Section included information about age, gender, nationality, marital status, level of education, year of experience in cleaning, and the type of hospital where the environmental staff works.

#### Clinical information

Section covered the current health status of the participant and the presence of any comorbidity.

#### Knowledge section

This section comprised five dimensions with a total of 26 items. These questions were designed to assess the cleaners' understanding of various aspects related to COVID-19, including its nature, symptoms, mode of transmission, prevention and treatment measures, risk factors, and cleaning and disinfection procedures. Participants responded to the questions using a true/false format, with the option of "do not know" also available. Each correct response was assigned a value of "1," while incorrect or "don't know" responses were given a value of "0." The Knowledge score was calculated by summing the scores for each item, resulting in a possible range of 0 to 26 points. The overall knowledge of the cleaners was categorized as good if the score fell between 60 and 100% (16–26 points) and poor if the score was below 60% (< 16 points), based on the modified Bloom's cut-off point [16].

#### Attitude section

To assess the attitudes of the cleaners, three dimensions were used. The first dimension focused on their attitudes towards health facilities, the second dimension examined their attitudes towards health authorities, and the final dimension targeted their attitudes towards cleaning and disinfection. Participants rated their agreement with the statements using a 3-point Likert scale, with "1" indicating disagreement and "3" indicating agreement. A score of 1 was assigned to responses indicating agreement, while responses indicating disagreement or neutrality received a score of 0. Similar to the knowledge section, the attitude score in each domain was categorized as positive if the score was between 60 and 100% and negative if the score was below 60%.

#### Practice section

This section comprised 21 items. Participants rated their practice behaviors using a 3-point Likert scale: "never," "occasionally," and "all the time." The scale aimed to capture specific information regarding the frequency of adopting infection prevention and control (IPC) measures by the cleaners. A score of 1 was given to responses indicating "all the time," while responses indicating "never" or "occasionally" received a score of 0. The practice score was also categorized using the original Bloom's cut-off point, with scores falling between 60 and 100% considered good and scores below 60% considered poor. Before the actual data collection period, the questionnaire was pre-tested on 5% of the sample. This allowed for the evaluation of the survey flow, functionality, and language.

### Calculation of sample size

The sample size was determined using the online RAOSOFT sample size calculator for web survey software (http://www.raosoft.com/samplesize.html). With an estimated population of 4,000 actively practicing housekeepers at the health facility level, a confidence level of 95%, and an absolute error of 5%, the minimum required sample size was calculated to be 351 participants.

### Data collection

An online questionnaire using Google Docs was distributed via email to directors of government-run and private hospitals across various regions in the country. Subsequently, designated focal persons within Lebanese hospitals were contacted by phone and informed about the survey and its purpose. After obtaining their agreement to participate, the survey link was shared with them via "WhatsApp". The respective hospitals then disseminated the survey link to their cleaners and housekeepers working in the health facilities through social media platforms, predominantly WhatsApp. The survey link clearly stated that all cleaners working in Lebanese hospitals who were capable of reading and writing in Arabic were eligible to participate. The link also provided a brief introduction to the background of the study, its objectives, and instructions for completing the questionnaire. Cleaners who were seriously ill or on annual leave during the data collection period were excluded from participation.

### Ethical approval

Each participant provided electronic informed consent before participating in the survey. Participation in the study was entirely voluntary, and all information collected was kept anonymous and treated with strict confidentiality. The study design ensured the adequate protection of participants, and the information presented did not pose any plausible harm or stigma to the participants. It is important to note that the study does not involve clinical data about patients and does not qualify as a clinical trial. Consequently, the study was exempted from requiring ethical approval by the Lebanese Ministry of Public Health.

### Data analysis

The collected data was exported from the Google Form to Microsoft Excel 2016 for cleaning and coding purposes. Data analysis was conducted using SPSS (Statistical Package for Social Sciences), version 22.0. The reliability of the knowledge, attitudes, and practice scales was assessed using Cronbach's alpha. Descriptive statistics were used to report categorical variables, including frequency and percentages. Single-item knowledge questions were presented as percentages of correct responses. Bivariate analysis was performed using chi-squared tests to examine the relationship between nominal variables. A significance level of *p*-value < 0.05 was considered statistically significant. Variables with a *p*-value < 0.2 in the bivariate analysis were included in a multivariable logistic regression model to identify factors associated with the dependent variable (good practice). The adequacy of the final logistic regression model was assessed using the Hosmer and Lemeshow test. The adjusted odds ratio and the corresponding 95% confidence intervals were reported.

## Results

### Baseline characteristics of the study participants

Table 1 presents the baseline characteristics of the survey participants. A total of 453 cleaners completed the survey, with females comprising 54.3% of the sample. Approximately one-third of the participants were in the age range of 21–30 years. The majority of respondents were Lebanese (82.34%), married (65.56%), and had a level of education up to a school degree or lower (68.65%). Most participants reported good health status (89.95%), while 27.59% had comorbidities. About two-thirds of the cleaners worked in private hospitals, and nearly half of them had three or more years of experience in cleaning health facilities. In terms of income, approximately 80.79% reported earning less than 1 Million Lebanese pounds.

**Table 1:** Baseline information of the hospital cleaners services staff, Lebanon 2020 (*N* = 453)

	*n*	%
**Gender**		
Male	207	45.70%
Female	246	54.30%
**Age**		
21–30 years	152	33.55%
31–40 years	146	32.23%
41–50 years	125	27.59%
More than 50 years	30	6.62%
**Nationality**		
Lebanese	373	82.34%
Other	80	17.75%
**Marital status**		
Married	297	65.56%
Others^a^	156	34.44%
**Educational level**		
Middle School degree or less	311	68.65%
Secondary or BT degree or more	142	31.35%
**Health status**		
Fair	21	4.64%
Average	25	5.52%
Good	407	89.85%
**Presence of comorbidities**		
No	328	72.41%
Yes	125	27.59%
**Type of hospital**		
Private	347	76.60%
Public	106	23.40%
**Income**		
Less than 1 Million L.L	366	80.79%
1–2 Millions L.L	84	18.54%
More than 2 Millions L.L	3	0.66%
**Years of experience in hospital cleaning**		
Less than 3 years	217	47.90%
3 years or more	236	52.10%
**Total**	**453**	**100%**

Others^a^ includes single, divorced and widowed

*N* Frequency, *%* Percentage

### Cleaners self-reported knowledge

**Fig. 1:**
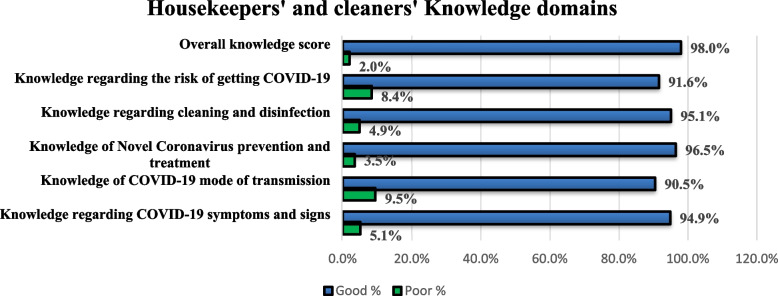
Knowledge domains of hospital cleaning services staff, Lebanon 2020

The knowledge scale demonstrated good reliability, as indicated by a Cronbach's alpha value of 0.782. Nearly 98% of the cleaners surveyed obtained a good overall knowledge score, indicating a strong understanding of the subject matter (≥ 60%). Figure 1 provides a visual representation of the cleaners' knowledge scores across different domains. The majority of respondents exhibited high levels of awareness and knowledge, with over 90% achieving good scores in all targeted knowledge domains, including the nature and symptoms of COVID-19, its mode of transmission, prevention and treatment, factors increasing the risk of infection, and decontamination procedures. While the majority of cleaners (90%) answered the majority of knowledge items correctly, some questions were less familiar and yielded the lowest correctness rates. For example, questions related to the transmission of COVID-19 through urine and feces of an infected person had a correct response rate of only 46.4%. Awareness regarding the availability of specific treatment for COVID-19 (66.9%) and the need to reduce the frequency of cleaning in the room of a COVID-19 patient (67.1%) was relatively lower. Similarly, items pertaining to the importance of rinsing surfaces after applying disinfectant (51.7%), and factors amplifying the risk of COVID-19 infection, such as virus concentration (69.3%) and the time elapsed since an infected person left the area (72.8%), were less well-known compared to other knowledge items (refer to Additional file 1: Appendix 1 for further details).

## Cleaners' attitudes

### Assessment of cleaners attitudes towards health facility

The attitude scale exhibited a good level of reliability, with a Cronbach's alpha value of 0.819. The majority of cleaners (87.7%) expressed agreement that patient safety is prioritized in the hospital where they work, and they believe that the hospital values their safety (90.7%). Furthermore, they reported that the hospital provides them with the necessary equipment and supplies for cleaning and disinfection (90.7%). Similarly, they perceived that infection prevention and control (IPC) measures are well implemented at the hospital level (87.2%), and the health facility has effectively fulfilled its role in raising awareness about the risk of COVID-19 transmission and its prevention (90.4%). Approximately half of the cleaners (47%) indicated that their hospital is continuously seeking innovative approaches to enhance hygiene practices. However, a notable proportion of cleaners (33.8%) felt that their hospital does not appreciate their extra efforts, and 29.8% believed that even if they perform their best, it will go unrecognized by the hospital. Similarly, around 28% of cleaners expressed that the hospital pays little attention to their concerns and disregards their complaints and requests (see Additional file 1: Appendix 2 for additional details).

### Cleaners' attitudes towards health authorities

Among the 453 participants, a substantial majority of cleaners, specifically 321 individuals (76.9%), expressed their belief that Lebanese health authorities have actively fulfilled their responsibilities in combating COVID-19, particularly within healthcare settings, by supplying personal protective equipment (PPE) and delivering training programs. Additionally, the majority of respondents (66.2%) expressed their confidence in the government's ability to successfully overcome the challenges posed by the COVID-19 pandemic (Additional file 1: Appendix 2).

### Cleaners' attitudes towards cleaning and disinfection

**Fig. 2:**
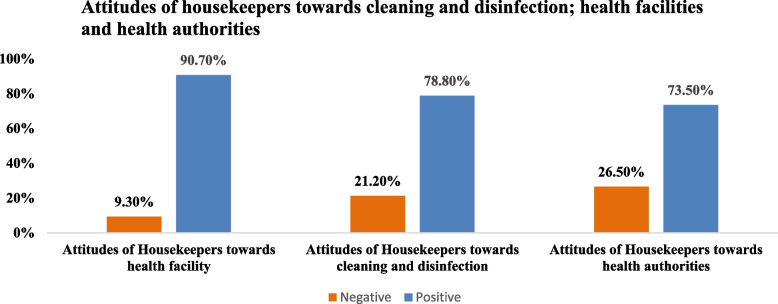
Attitudes of hospital cleaning services staff towards health facilities, health authorities, and cleaning procedures in Lebanon: 2020

The survey results revealed that a substantial majority of cleaners, exceeding 85%, recognized the effectiveness of essential preventive measures in combating COVID-19, including regular hand washing, proper utilization of personal protective equipment (PPE), and thorough cleaning and disinfection of surfaces. These measures were perceived as crucial for eliminating the virus and preventing infection. Figure 2 provides a graphical representation of the attitude scores of the cleaners. The findings indicate that the majority of cleaners exhibited a positive attitude towards health facilities, health authorities, and the implementation of infection prevention and control (IPC) precautions (Additional file 1: Appendix 2).

## Cleaners' practices

Table 2 presents the items related to cleaners' practices. Upon calculating Cronbach's alpha, the practice scale demonstrated good reliability (α = 0.713). The findings indicate that the majority of cleaners consistently adhered to infection prevention and control (IPC) standard precautions. This included practicing hand hygiene (90.7%), maintaining social distancing (75.7%), avoiding crowded places (85.7%), wearing face masks at the hospital (91.4%), refraining from touching surfaces and then their eyes or faces (92.5%), and observing proper cough and sneeze etiquette (94%). Additionally, 92.4% of the cleaners reported compliance with the prevention measures recommended by the Ministry of Public Health (MOPH).

**Table 2:** Self-reported practices of hospital cleaning services staff during COVID19 pandemic in Lebanon, November 2020

Practices	Never	Occasionally	All the times
	***n*(%)**	***n*(%)**	***n*(%)**
Washing hands with soap and water, and also using disinfectants regularly	6(1.3%)	35(1.3%)	412(90.9%)
Maintaining social distance with everyone in the hospital (1.5 m)	3(0.7%)	107(23.6%)	343(75.7%)
Wearing a face mask all the time at the hospital	12(2.6%)	27(6%)	414(91.4%)
Avoiding the presence in crowded places	9(2%)	56(12.4%)	388(85.7%)
Avoiding touching surfaces than touching eyes or faces	9(2%)	25(5.5%)	419(92.5%)
Adhere to the prevention measures requested by MOPH	6(1.3%)	29(6.4%)	418(92.3%)
Respecting cough and sneeze etiquette	3(0.7%)	24(5.3%)	426(94%)
Checking the availability of the needed cleaning supplies with my supervisor before starting	3(0.7%)	53(11.7%)	397(87.6%)
Cleaning and disinfecting common areas at hospitals (such as rest rooms, halls, reception, corridors and lifts)	3(0.7%)	46(10.2%)	404(89.2%)
Cleaning and disinfecting surfaces and objects that are frequently touched, such as handles, elevator buttons, handrails, doorknobs and dispensers	3(0.7%)	34(7.5%)	416(91.8%)
Keeping the room where disinfectant solution is prepared used aerated and Labelling the prepared solution of disinfectant	12(2.6%)	61(14.1%)	377(83.2%)
Following the manufacturer’s instructions to ensure that disinfectants are prepared and handled safely	9(2%)	31(6.8%)	413(91.2%)
Wearing appropriate PPE when visiting COVID-19 patient room	3(0.7%)	21(4.6%)	429(94.7%)
Donning and doffing PPEs appropriately	5(1.1%)	33(7.3%)	415(91.6%)
Following regular training on IPC including PPEs donning and doffing	11(2.4%)	44(9.7%)	398(87.9%)
I wash my hands before and after wearing PPEs	3(0.7%)	26(5.7%)	424(93.6%)
Handling laundry carefully to mitigate the risk of potential transmission	3(0.7%)	23(5%)	427(94.3%)
Putting textiles, linens, and clothes in special, marked laundry bags	21(4.6%)	28(6.2%)	404(89.2%)
Washing laundry in warm cycles (60-90ºC) with the usual detergents	21(4.6%)	53(11.7%)	379(83.7%)
Placing disposable items (hand towels, gloves, medical masks, tissues) in a container with a lid and following hospital action plan and national regulations for waste management	6(1.3%)	24(5.3%)	423(93.4%)
Following COVID-29 news	30(6.6%)	135(29.8%)	288(63.6%)

Regarding cleaning and disinfection practices, 87.6% of the surveyed cleaners confirmed that they always checked with their supervisor for the availability of necessary cleaning supplies before commencing work. Over 80% of them ensured proper ventilation in rooms and consistently followed the manufacturer's instructions to ensure safe handling and preparation of disinfectants. Furthermore, approximately 90% of the cleaning staff reported regular cleaning and disinfection of common areas in hospitals, as well as high-touch surfaces such as handles, elevator buttons, handrails, and doorknobs. Moreover, a significant majority (94.3%) demonstrated careful and appropriate handling of laundry to minimize the risk of potential transmission. Specifically, 89.2% used designated, labeled laundry bags for textiles, linens, and clothes, while 83.7% washed laundry in warm cycles (60-90ºC) using standard detergents.

Concerning the use of personal protective equipment (PPE), 94.7% of cleaners reported consistent usage of appropriate PPE when entering the room of COVID-19 patients for cleaning purposes. Additionally, 91.6% of them confirmed proper donning and doffing of PPE. Furthermore, more than 90% of the cleaners consistently followed recommended hand hygiene practices before and after wearing PPE or engaging in cleaning activities. The majority of respondents (87.9%) also attended regular training sessions on IPC during the COVID-19 pandemic. In terms of waste management, 93.4% of cleaners reported efficient handling of waste by placing disposable items (hand towels, gloves, medical masks, and tissues) in containers with lids, and they followed both the hospital's waste management plan and national regulations. However, only 63.6% of them consistently stayed updated with COVID-19 news.

## Sources of information

**Fig. 3:**
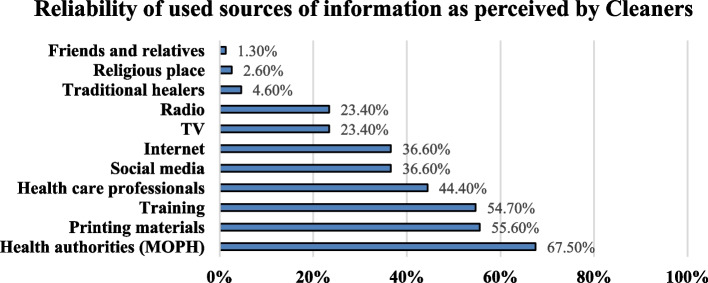
Perceived reliability of COVID-19 information sources among hospital cleaning services staff: Lebanon, 2020

Cleaners reported that the most frequently accessed sources of information were health care professionals (66.8%), health authorities (66.2%), and social media (61.6%) (Additional file 1: Appendix 3). However, when it came to reliability, the cleaners ranked the Ministry of Public Health (MOPH) as the most trustworthy source (67.5%), followed by printed materials (55.6%) and trainings (54.7%) (Fig. 3).

## Factors associated with cleaners' good practices

Table 3 presents the results of the multivariable logistic regression analysis examining the factors associated with cleaners' adoption of good practices. The findings indicate that gender, marital status, and the presence of comorbidities were not significantly associated with good practices. However, age was found to be a significant factor. Cleaners aged over 40 years were less likely to adopt good practices compared to their younger counterparts, with an adjusted odds ratio (aOR) of 0.712 (95% CI: 0.202 to 0.851). On the other hand, cleaners with a secondary degree education or higher were 1.869 times more likely to engage in good practices compared to those with the lowest educational level (aOR = 1.869, 95% CI: 1.271 to 3.045).

**Table 3:** Multivariable logistic regression analysis of factors associated with good practices among hospital cleaning services staff: Lebanon, 2020

	**Poor practices**	**Good practices**	***P*-value**	**aOR**	**95% C.I.for aOR**	
	**n(%)**	**n(%)**			**Lower**	**Upper**
**Gender**			0.18			
Male	19(9.2%)	188(90.8%)				
Female	28(11.4%)	218(88.6%)				
**Age**			** < 0.001**			
21–30 years	1(0.7%)	151(99.3%)		1.000		
31–40 years	8(5.5%)	138(94.5%)		0.851	0.605	1.576
More than 40 years	38(24.5%)	117(75.5%)		0.712	0.202	0.851
**Marital status**			0.208			
Single	5(4.5%)	106(95.5%)				
Others (single, separated or widowed)	15(9.7%)	141(90.3%)				
**Educational level**			**0.023**			
Middle School degree or less	39(12.5%)	272(87.5%)		1.000		
Secondary or BT degree or more	8(5.7%)	134(94.3%)		1.869	1.271	3.045
**Presence of comorbidities**			0.251			
No	37(11.3%)	291(88.7%)				
Yes	10(8%)	115(92%)				
**Type of hospital**			** < 0.001**			
Public	27(25.5%)	79(74.5%)		1.000		
Private	20(5.8%)	327(94.2%)		2.083	1.340	3.436
**Years of experience in hospital cleaning**			** < 0.001**			
Less than 3 years	40(18.43%)	177(81.57%)		1		
3 years and more	7(3%)	229(97%)		2.942	2.214	3.747
**Knowledge regarding COVID-19 symptoms**			**0.006**			
Poor	10(43.5%)	13(56.5%)		1.000		
Good	37(8.6%)	393(91.4%)		2.076	1.895	3.463
**Knowledge of COVID-19 mode of transmission**			0.833			
Poor	6(14%)	37(86%)				
Good	41(10%)	369(90%)				
**Knowledge of COVID-19 prevention and treatment**			**0.019**			
Poor	6(37.5%)	10(62.5%)		1		
Good	41(9.4%)	396(90.6%)		2.717	1.594	4.051
**Knowledge of cleaning and disinfection**			**0.031**			
Poor	6(27.3%)	16(72.7%)				
Good	41(9.5%)	390(90.5%)		2.934	1.699	4.126
**Knowledge about the risk factors of getting COVID-19**			**0.004**			
Poor	10(26.3%)	28(73.7%)		1		
Good	37(8.9%)	378(91.1%)		1.937	1.569	3.150
**Attitudes of Housekeepers towards health facility**			** < 0.001**			
Negative	16(35.6%)	29(64.4%)		1		
Positive	31(7.6%)	377(92.4%)		2.481	1.591	4.388
**Attitudes of Housekeepers towards cleaning and disinfection**			** < 0.001**			
Negative	23(26.1%)	65(73.9%)		1		
Positive	24(6.6%)	341(93.4%)		3.939	1.819	5.126
**Attitudes of Housekeepers towards health authorities**			**0.021**			
Negative	27(32.1%)	57(67.9%)		1		
Positive	20(5.4%)	349(94.6%)		2.303	1.106	3.863

*C.I* Confidence interval, *aOR* Adjusted Odds Ratio

Furthermore, working in private hospitals (aOR = 2.083, 95% CI: 1.340 to 3.436) and having more than 3 years of experience in hospital cleaning (aOR = 2.942, 95% CI: 2.214 to 3.747) were positively associated with the adoption of good practices. In terms of knowledge, cleaners with good knowledge levels in various domains related to COVID-19, including its nature, prevention and treatment, cleaning and disinfection, and factors affecting the risk of COVID-19, were more likely to exhibit good practices (aOR range: 1.397 to 2.934, all *p* < 0.05).

Moreover, cleaners with a positive attitude towards health facilities (aOR = 2.481, 95% CI: 1.591 to 4.388) and a positive attitude towards health authorities and the government (aOR = 2.303, 95% CI: 1.106 to 3.863) were more likely to demonstrate good practices. Lastly, participants with a positive attitude towards cleaning and disinfection were 3.939 times more likely to implement good practices during their work.

## Discussion

The COVID-19 pandemic has brought attention to the significant importance of hygiene and infection prevention, particularly in healthcare settings. To address this, the CDC and WHO have established extensive guidelines and recommendations for healthcare facilities to implement effective infection control practices [14, 16]. These measures encompass various aspects such as maintaining proper hand hygiene, ensuring the appropriate use of personal protective equipment (PPE), following disinfection and cleaning protocols, practicing respiratory hygiene, and implementing patient isolation procedures. As a result, the pandemic has highlighted the often-underestimated contributions of hospital cleaners, whose essential role in upholding cleanliness and hygiene within healthcare settings has become more evident. To our knowledge, this is the first nationwide study conducted in Lebanon that aims to investigate the knowledge, attitudes, and practices of environmental cleaning staff regarding cleaning and disinfection during the COVID-19 pandemic. By gaining insights from cleaners, we can identify areas for improvement and target interventions to address any gaps or weaknesses.

Our study revealed several key findings. Firstly, the majority of surveyed cleaners (98%) demonstrated a good overall knowledge score, indicating a high level of awareness across various knowledge domains related to COVID-19. This encompassed understanding the nature and symptoms of the virus, its modes of transmission, preventive measures, treatment options, risk factors, and decontamination procedures. These findings align with a similar study conducted among healthcare workers in Ethiopia, where 84.7% displayed good knowledge levels [18]. A study conducted in Addis Ababa yielded similar findings [19]. However, our results regarding knowledge scores surpassed those reported in other studies conducted in hospitals in Palestine (53.9%) and Iran (57%) [20, 21]. This discrepancy may be attributed to variations in sample size, sociodemographic factors, and the specific items used to assess knowledge. While numerous studies have examined various aspects of this subject among healthcare workers (HCWs), none of them have specifically addressed the knowledge and application of infection control principles and cleaning practices among environmental services staff members. Consequently, there is a notable gap in the literature, making direct comparisons difficult.

Nevertheless, certain pieces of information remained less recognized by the cleaners, such as the potential transmission of COVID-19 through urine or feces and the availability of specific treatments for the virus. It is worth noting that although COVID-19 has been detected in stool samples and, rarely, in urine, current evidence does not support fecal or wastewater exposure as a primary route of transmission for the virus [22]. Furthermore, it is important to note that the availability of specific treatments for COVID-19 is time-sensitive and subject to ongoing trials. Similarly, not all cleaners were aware of the need to reduce the frequency of cleaning in COVID-19 patient rooms. While enhancing hygiene practices during the pandemic is crucial, it is also necessary to strike a balance between effective cleaning and minimizing the risk of COVID-19 exposure. Compared to other areas of knowledge, cleaners exhibited a lower level of awareness regarding factors that amplify the risk of COVID-19 transmission. Therefore, raising awareness about these factors is essential in empowering cleaners to effectively prevent infections.

Likewise, the majority of respondents expressed a strong agreement on questions related to the importance of cleaning in reducing infections for patients and their families. Cleaners demonstrated a clear understanding of their responsibilities and assigned a high priority to patient safety within their organization.

In terms of attitude, most cleaners exhibited a positive outlook towards health facilities. They believed that hospitals prioritize patient safety and the well-being of their staff. Cleaners acknowledged that hospitals provided them with the necessary equipment and supplies for cleaning and disinfection, implemented infection prevention and control measures, and offered education on the risk of contracting COVID-19. This positive attitude can be attributed to their good levels of knowledge, as supported by findings from a study conducted in Iran [23]. However, it is important to recognize that cleaners' attitudes and perceptions about their work can influence their motivation and the effectiveness of their cleaning practices. Therefore, understanding and addressing these attitudes and beliefs are crucial for continual improvement of environmental cleaning services [7].

Similarly, cleaners demonstrated a strong consensus on the importance of cleaning in preventing infections, their understanding of their role expectations, and their prioritization of patient safety within their organization. These findings align with studies conducted in Canada and the United States, which highlighted the recognition among environmental services workers (ESWs) of the significance of their work in ensuring patient safety. Additionally, cleaners displayed a sense of dedication and pride in their work [24]. Notably, a majority of participants in our study emphasized the importance of receiving feedback on their performance. Unfortunately, most cleaners reported a lack of regular feedback regarding their work.

However, our findings also revealed that many cleaners do not feel appreciated by the hospital and other healthcare workers for their additional efforts. More than a quarter of them believed that the hospital showed little interest in them, neglected their well-being, and disregarded their complaints and requests. The lack of recognition and appreciation for their efforts may lead to demotivation among some cleaners, resulting in a decline in their performance. Similar findings were reported in a study conducted among environmental services workers in New York [24]. Therefore, the perceived lack of organizational support, feedback, and investment in cleaning resources should not be underestimated. Understanding the factors that influence cleaning performance is crucial for developing strategies to enhance healthcare cleanliness and reduce the risk of infection transmission.

It is worth noting that most cleaners expressed positive attitudes towards Lebanese health authorities and believed that the government has effectively fulfilled its role in combating COVID-19, especially within healthcare settings. These positive attitudes and high confidence in controlling COVID-19 can be attributed to the Lebanese government's proactive response, including implementing strict control measures and precautions such as lockdowns, flight suspensions, and restrictions on public gatherings. Similarly, the majority of cleaners demonstrated positive attitudes towards infection prevention and control (IPC) measures, including handwashing, wearing personal protective equipment (PPE), and surface decontamination. They recognized these measures as effective in reducing the risk of COVID-19 infection.

Based on the responses of cleaners, a significant majority consistently adhered to IPC standards, including practicing hand hygiene, maintaining social distancing, avoiding crowded places, wearing face masks at the hospital, refraining from touching surfaces and then their faces, and following proper cough and sneeze etiquette. Furthermore, 92.4% of cleaners reported compliance with the prevention measures recommended by the Ministry of Public Health (MOPH). This high level of self-reported compliance aligns with the findings of a previous study conducted among other healthcare workers (HCWs) [25]. However, other studies that used observation for data collection reported lower compliance rates. However, studies that utilized observation-based data collection methods have reported lower compliance rates [26].

Regarding cleaning and disinfection practices, most surveyed cleaners followed recommended protocols. They ensured the availability of necessary cleaning supplies and equipment before starting their work, maintained proper ventilation in the rooms, and followed manufacturer's instructions for safe handling of disinfectants. They diligently cleaned and disinfected common areas, frequently touched objects, and handled laundry with care. When it came to personal protective equipment (PPE), over 90% of cleaners stated that they always wore appropriate PPE when cleaning COVID-19 patient rooms or units, and they followed proper donning and doffing procedures along with practicing hand hygiene. Additionally, a significant proportion of cleaners implemented efficient waste management practices in accordance with the hospital's action plan and national regulations. However, it is important to note that such statements may be influenced by social desirability bias, emphasizing the need for field studies that closely observe actual practices.

Our findings also revealed that the majority of cleaners regularly attended training sessions on IPC during the COVID-19 pandemic. This finding helps explain the high levels of knowledge and adherence observed among the surveyed cleaners. Training sessions focused on current guidelines likely enhanced their understanding of fundamental practice standards and facilitated consistent implementation. Moreover, up-to-date knowledge and skills regarding cleaning and disinfection likely increased cleaners' confidence in complying with recommended guidelines.

More than half of the cleaners reported consistently following COVID-19 news, with a notable finding that social media was the most commonly used source of information. However, it is important to note that while social media platforms provide easy access to information, they can also be a breeding ground for fake news and panic [27]. Therefore, it is highly recommended for cleaners to seek information from scientific and reputable sources. Interestingly, the cleaners ranked the Ministry of Public Health (MOPH) as the most reliable source of information, followed by printed materials. This highlights the significance of government involvement in providing real-time emergency information during infectious disease outbreaks, as it contributes to promoting engagement in protective behaviors [28].

Gender, marital status, and the presence of comorbidities were not found to be associated with the adoption of good practices. However, our findings indicated a negative association between age and good practices, with cleaners aged 31 and above being less likely to adopt good practices compared to those aged 21–30 years. It is worth noting that previous studies have yielded inconsistent results regarding the impact of age [29–31].

Furthermore, our results revealed that work experience was a strong predictor of good practices. Cleaners with more than three years of experience were four times more likely to exhibit good practices in cleaning and disinfection compared to their counterparts with less than three years of experience. As the number of years of practice increases, cleaners are exposed to hazards repeatedly and gain valuable experience through working with senior staff.

In terms of educational level, cleaners with a higher level of education were more likely to adopt good practices compared to those with lower educational levels. This finding aligns with a study conducted in Ethiopia, which found that a higher educational level was associated with engaging in infection prevention and control activities [32]. One possible explanation for this association is that cleaners with higher educational levels may have better access to local and international information sources and training platforms, enabling them to adopt good practices.

Additionally, this study revealed that cleaners working in private hospitals were ten times more likely to adopt good practices. This finding is consistent with a study conducted in Bangladesh among healthcare workers [33]. Private hospitals may have implemented regular administrative supervision and monitoring of cleaners, along with intensive training on cleaning and disinfection, proper use of personal protective equipment (PPE), and waste management. The financial resources generated by private hospitals may contribute to ensuring better quality services compared to government hospitals.

Knowledge serves as the foundation for effective practices, and in this study, cleaners with good knowledge about COVID-19 were more likely to adopt good practices in infection prevention and control. Therefore, knowledge plays a crucial role in promoting preventive measures and enhancing good practices in the fight against the disease.

Furthermore, attitude has long been recognized as a key factor influencing people's behavior [34, 35]. Cleaners who held positive attitudes towards health facilities or health authorities were more likely to exhibit good practices. Having a positive attitude towards cleaning and disinfection emerged as a significant predictor for good practices in the workplace. Cleaners' attitudes and beliefs regarding the importance of cleaning and disinfecting can impact their motivation to clean and, consequently, the effectiveness of their efforts [36]. Numerous studies have demonstrated successful infection control outcomes following the implementation of enhanced cleaning and disinfection approaches [34].

## Implications of the study

This study provides valuable insights that have practical implications for improving cleaning practices, enhancing public health outcomes, and guiding future research efforts in this domain.

As for its implications in clinical practice, the study's findings can be utilized to develop targeted training programs for cleaners, aimed at improving their knowledge and adherence to recommended cleaning protocols. By enhancing their understanding and practices, the risk of infection transmission in healthcare settings can be effectively reduced.

In terms of public health, the study underscores the importance of fostering a supportive work environment for cleaners. Recognizing the impact of their attitudes towards health facilities and authorities, efforts should be made to create an environment that motivates and empowers cleaners to maintain cleanliness and prevent infections effectively.

Finally, this study contributes to the existing body of literature on the experiences of cleaners during the COVID-19 pandemic, specifically within the unique context of Lebanon. The insights gained from this study can inform further research endeavors aimed at comprehensively understanding the factors that influence cleaners' knowledge, attitudes, and practices. Such research can facilitate the identification and implementation of effective strategies to enhance cleaning practices in healthcare facilities.

## Limitations of study

Our study has several limitations that should be acknowledged. Firstly, due to its cross-sectional design, we cannot establish a temporal relationship between the outcome and predictor variables. Additionally, the study is susceptible to social desirability bias, which may lead to over- or underestimation of the findings. While validated questions were used in the questionnaire, some respondents had a different first language than Arabic, resulting in difficulties comprehending the Arabic version of the questionnaire for those who were not fluent in Arabic. This hindered their participation in the study. Future studies or hospitals wishing to assess the knowledge of environmental services staff members may consider using verbal surveys. The positioning of questions related to attitudes, roles of cleaning, and perceived organizational support at the end of the questionnaire, which primarily consisted of questions for other purposes, may have influenced responses to these specific questions. In certain hospitals, environmental services staff members expressed cynicism when asked about their attitudes and perceived organizational support. Despite reassurances about data confidentiality, some respondents were concerned about their responses being identifiable by the hospital, especially regarding perceived organizational support. For future studies, it is recommended to separate the assessment of knowledge and practice from attitudes and perceived organizational support.

## Conclusion

Cleaning staffs are on the front lines of fighting this global pandemic. Our results indicate that environmental services staff members have a high level of knowledge and expressed positive attitudes towards health facilities and authorities. They are also aware of the importance of their role. Nevertheless, there seems to be a perceived lack of support, feedback, and investment from the organization in terms of cleaning resources. It is important not to disregard the attitudes of the environmental services staff members, and it is essential to understand the factors that influence their performance in order to develop targeted interventions that enhance hospital cleaning and minimize the risk of infection transmission. The findings of this study will benefit future planning if another outbreak wave occurs.

## Supplementary Information

**Additional file 1: Appendix 1.** Hospital cleaning services staff responses to knowledge items (*N*= 453). **Appendix 2.** Attitudes of housekeepers and cleaners towards health facility, health authorities and cleaning. **Appendix 3. **Sources of information reported by cleaners to get information about COVID-19.

**Additional file 2.**

## Data Availability

The datasets generated during the current study are not publicly available but are available from the corresponding author on reasonable request.

## Abbreviations

MOPH: Ministry of Public Health
KAP: Knowledge, attitudes and practices
HCWs: Health care workers
COVID-19: Coronavirus disease 2019
aOR: Adjusted Odds Ratio
SPSS: Statistical Package for Social Sciences
