# Supplementary material for: Unveiling the unknown: first comprehensive assessment of the knowledge, attitudes and practices of hospital cleaning services staff regarding COVID-19 in Lebanon during the pandemic
Source: Arch Public Health. 2023 Jul 17;81:134. doi: 10.1186/s13690-023-01149-5 (PMC10353244; doi:10.1186/s13690-023-01149-5)
Supplement: Supplementary file 1 — Additional file 1: Appendix 1. Hospital cleaning services staff responses to knowledge items (N= 453). Appendix 2. Attitudes of housekeepers and cleaners towards health facility, health authorities and cleaning. Appendix 3. Sources of information reported by cleaners to get information about COVID-19. [file 13690_2023_1149_MOESM1_ESM.docx]

**Appendix 1: Hospital cleaning services staff responses to knowledge items (N= 453)**

|  | **Correct** | **Incorrect** |
| --- | --- | --- |
|  | **n(%)** | **n(%)** |
| **Domain1: Knowledge regarding COVID-19 symptoms and signs** | | |
| The main clinical symptoms of COVID-19 are fever, fatigue, dry cough and myalgia | 421(92.9%) | 32(7.1%) |
| All persons with COVID-19 will develop severe cases and might die | 309(68.2%) | 144(31.8%) |
| Only elderly and those having chronic illnesses are more likely to be severe cases | 422(93.2%) | 31(6.9%) |
| **Domain 2: Knowledge of COVID-19 mode of transmission** |  |  |
| COVID-19 can be transmitted from person to person via cough and sneezes | 438(96.7%) | 15(3.3%) |
| COVID-19 can be transmitted by urine and feces of an infected person | 210(46.4%) | 173(53.7%) |
| COVID19 can be transmitted by kissing, hugging, shaking hand with an infected person | 421(92.9%) | 32(7.1%) |
| An infected person with COVID-19 can be asymptomatic and spread the infection to others | 419(92.5%) | 34(7.5%) |
| **Domain 3: Knowledge of Novel Coronavirus prevention and treatment** | | |
| COVID-19 can be prevented by regular washing hands with soap and water and also by using sanitizers | 438(96.7%) | 15(3.3%) |
| COVID-19 can be prevented by using a mask | 435(96%) | 18(4%) |
| To date, there is no specific treatment for COVID-19 | 303(66.9%) | 150(33.1%) |
| Any COVID-19 exposure should be immediately reported to the national call center | 422(93.2%) | 31(6.9%) |
| The number of people visiting the patient room (in contact with the patient) should be minimized | 416(91.8%) | 37(8.2%) |
| The time spent by the cleaner in the room of the COVID-19 patient should be reduced | 387(85.4%) | 66(14.6%) |
| **Domain 4: Knowledge regarding cleaning and disinfection** |  |  |
| Any surfaces that have been in contact with the ill person(s), such as toilet, handwashing basins, and baths, should be cleaned, then disinfected | 434(95.8%) | 19(4.2%) |
| Cleaning should always be performed first, followed by disinfection for surfaces and items touched by COVID-19 case. | 408(90.1%) | 45(9.9%) |
| If the use of bleach is not suitable, or potentially damaging to the surface (on devices like telephones and remote-control equipment), 70% alcohol could be used. | 326(72%) | 127(28%) |
| When preparing bleach solution, concentration should be monitored | 422(93.2%) | 31(6.9%) |
| Rinsing surface is required after the application of disinfectant | 254(56.1%) | 199(43.9%) |
| While cleaning, using disposable cleaning materials whenever possible | 399(88.1%) | 54(11.9%) |
| Linen should be bagged before being removed from the room but does not require special laundering | 435(96%) | 18(3.9%) |
| The frequency of cleaning of the room of COVID-19 patient should be reduced | 304(67.1%) | 149(32.8%) |
| Cleaning staff should wear PPEs when visiting the room of COVID-19 patient | 418(92.3%) | 35(7.7%) |
| **Domain 5: Knowledge regarding the risk of getting COVID-19** |  |  |
| The risk of COVID-19 infection depends on the type of surface where the virus is present | 353(77.9%) | 100(22.1%) |
| The risk of COVID-19 infection depends on the concentration of virus | 314(69.3%) | 139(30.7%) |
| The risk of COVID-19 infection depends time since the infected person left the place | 330(72.8%) | 123(27.2%) |
| The risk of COVID-19 infection depends time spend by the infected person in this place | 386(85.2%) | 67(14.8%) |

**Appendix 2:**

**Attitudes of housekeepers and cleaners towards health facility, health authorities and cleaning**

|  | **Disagree** | **Neutral** | **Agree** |
| --- | --- | --- | --- |
|  | **n(%)** | **n(%)** | **n(%)** |
| Patient safety is a priority in this hospital | 18(3.9%) | 24(5.4%) | 411(87.7%) |
| The hospital is always trying to find new ways to improve hygiene | 50(11.1%) | 190(41.9%) | 213(47%) |
| The hospital provides me by all the equipment needed for cleaning and sterilization | 15(3.3%) | 27(6%) | 411(90.7%) |
| The hospital does not appreciate any further effort exert from my part | 242(53.4%) | 58(12.8%) | 153(33.8%) |
| The hospital ignores any complaints or requests from my part | 287(63.4%) | 63(13.9%) | 103(22.8%) |
| The hospital really cares about my safety and my health | 21(4.65%) | 21(4.65%) | 411(90.7%) |
| Even if you do my best, the hospital will not appreciate it | 252(55.6%) | 66(14.6%) | 135(29.8%) |
| The hospital takes care of my general satisfaction at work | 72(13.6%) | 82(18.1%) | 309(68.2%) |
| The hospital does not take much interest in me | 253(55.9%) | 77(17%) | 123(27.1%) |
| I find it easy to ask my supervisor if help or advice are needed | 13(2.9%) | 17(3.8%) | 423(93.3%) |
| I think infection prevention measures are well implemented at hospital | 21(4.6%) | 37(8.2%) | 395(87.2%) |
| I think the hospital has done its part in raising awareness about the risk of catching COVID-19 and how to prevent it | 18(4%) | 30(6.6%) | 405(90.4%) |
| **Attitudes toward cleaning and disinfection** | | | |
| I believe that cleaning and disinfecting surfaces is effective for eliminating COVID-19 | 3(0.7%) | 61(13.5%) | 389(85.8%) |
| I think that PPEs can protect me from catching COVID-19 | 18(4%) | 39(8.6%) | 396(87.4%) |
| I believe that regular hand washing could prevent the COVID-19 infection | 14(3.1%) | 39(8.6%) | 400(88.3%) |
| **Attitudes towards health authorities** | | | |
| I think that MOPH has played her preventive role in the hospital (providing PPEs...) | 50(11.1%) | 82(18.1%) | 321(76.9%) |
| I have confidence that Lebanon can win the battle against the COVID-19 | 74(16.4%) | 79(17.4%) | 300(66.2%) |

**Appendix 3:**

**Sources of information reported by cleaners to get information about COVID-19**
