# Supplementary material for: Unveiling the unknown: first comprehensive assessment of the knowledge, attitudes and practices of hospital cleaning services staff regarding COVID-19 in Lebanon during the pandemic
Source: Arch Public Health. 2023 Jul 17;81:134. doi: 10.1186/s13690-023-01149-5 (PMC10353244; doi:10.1186/s13690-023-01149-5)
Supplement: Supplementary file 2 — Additional file 2. [file 13690_2023_1149_MOESM2_ESM.docx]

**Questionnaire__KAP cleaners**

| **Baseline information of participants** |
| --- |
| **Gender** |
| Male |
| Female |
| **Age** |
| 21-30 years |
| 31-40 years |
| 41-50 years |
| More than 50 years |
| **Nationality** |
| Lebanese |
| Other |
| **Marital status** |
| Married |
| Others* |
| **Educational level** |
| Middle School degree or less |
| Secondary or BT degree or more |
| **Health status** |
| Fair |
| Average |
| Good |
| **Presence of comorbidities** |
| No |
| Yes |
| **Type of hospital** |
| Private |
| Public |
| **Income** |
| Less than 1 Million L.L |
| 1-2 Millions L.L |
| More than 2 Millions L.L |
| **Years of experience in hospital cleaning** |
| Less than 3 years |
| 3 years or more |
| *Others^*^ includes single, divorced and widowed* |

**Sources of information used by Cleaners**

**Yes No**

Healthcare professionals

Health authorities (MOPH)

Social media

Internet

Training

TV

Friends and relatives

Printing materials

Radio

Traditional healers

Religious place

**Reliability of used sources of information**

**Yes No**

Healthcare professionals

Health authorities (MOPH)

Social media

Internet

Training

TV

Friends and relatives

Printing materials

Radio

Traditional healers

Religious place

| **Knowledge items** |  |  |
| --- | --- | --- |
|  | **Correct** | **Incorrect** |
| The main clinical symptoms of COVID-19 are fever, fatigue, dry cough and myalgia |  |  |
| All persons with COVID-19 will develop severe cases and might die |  |  |
| Only elderly and those having chronic illnesses are more likely to be severe cases |  |  |
| COVID-19 can be transmitted from person to person via cough and sneezes |  |  |
| COVID-19 can be transmitted by urine and feces of an infected person |  |  |
| COVID19 can be transmitted by kissing, hugging, shaking hand with an infected person |  |  |
| An infected person with COVID-19 can be asymptomatic and spread the infection to others |  |  |
| COVID-19 can be prevented by regular washing hands with soap and water and also by using sanitizers |  |  |
| COVID-19 can be prevented by using a mask |  |  |
| To date, there is no specific treatment for COVID-19 |  |  |
| Any COVID-19 exposure should be immediately reported to the national call center |  |  |
| The number of people visiting the patient room (in contact with the patient) should be minimized |  |  |
| The time spent by the cleaner in the room of the COVID-19 patient should be reduced |  |  |
| Any surfaces that have been in contact with the ill person(s), such as toilet, handwashing basins, and baths, should be cleaned, then disinfected |  |  |
| Cleaning should always be performed first, followed by disinfection for surfaces and items touched by COVID-19 case. |  |  |
| If the use of bleach is not suitable, or potentially damaging to the surface (on devices like telephones and remote-control equipment), 70% alcohol could be used. |  |  |
| When preparing bleach solution, concentration should be monitored |  |  |
| Rinsing surface is required after the application of disinfectant |  |  |
| While cleaning, using disposable cleaning materials whenever possible |  |  |
| Linen should be bagged before being removed from the room but does not require special laundering |  |  |
| The frequency of cleaning of the room of COVID-19 patient should be reduced |  |  |
| Cleaning staff should wear PPEs when visiting the room of COVID-19 patient |  |  |
| The risk of COVID-19 infection depends on the type of surface where the virus is present |  |  |
| The risk of COVID-19 infection depends on the concentration of virus |  |  |
| The risk of COVID-19 infection depends time since the infected person left the place |  |  |
| The risk of COVID-19 infection depends time spend by the infected person in this place |  |  |

| **Attitudes of housekeepers and cleaners towards health facility, health authorities and cleaning** | | | |
| --- | --- | --- | --- |
|  | **Disagree** | **Neutral** | **Agree** |
| Patient safety is a priority in this hospital |  |  |  |
| The hospital is always trying to find new ways to improve hygiene |  |  |  |
| The hospital provides me by all the equipment needed for cleaning and sterilization |  |  |  |
| The hospital does not appreciate any further effort exert from my part |  |  |  |
| The hospital ignores any complaints or requests from my part |  |  |  |
| The hospital really cares about my safety and my health |  |  |  |
| Even if you do my best, the hospital will not appreciate it |  |  |  |
| The hospital takes care of my general satisfaction at work |  |  |  |
| The hospital does not take much interest in me |  |  |  |
| I find it easy to ask my supervisor if help or advice are needed |  |  |  |
| I think infection prevention measures are well implemented at hospital |  |  |  |
| I think the hospital has done its part in raising awareness about the risk of catching COVID-19 and how to prevent it |  |  |  |
| I believe that cleaning and disinfecting surfaces is effective for eliminating COVID-19 |  |  |  |
| I think that PPEs can protect me from catching COVID-19 |  |  |  |
| I believe that regular hand washing could prevent the COVID-19 infection |  |  |  |
| I think that MOPH has played her preventive role in the hospital (providing PPEs...) |  |  |  |
| I have confidence that Lebanon can win the battle against the COVID-19 |  |  |  |

| **Practices items** |  |  |  |
| --- | --- | --- | --- |
| **Practices** | **Never** | **Occasionally** | **All the times** |
|  |  |  |  |
| Washing hands with soap and water, and also using disinfectants regularly |  |  |  |
| Maintaining social distance with everyone in the hospital (1.5 m) |  |  |  |
| Wearing a face mask all the time at the hospital |  |  |  |
| Avoiding the presence in crowded places |  |  |  |
| Avoiding touching surfaces than touching eyes or faces |  |  |  |
| Adhere to the prevention measures requested by MOPH |  |  |  |
| Respecting cough and sneeze etiquette |  |  |  |
| Checking the availability of the needed cleaning supplies with my supervisor before starting |  |  |  |
| Cleaning and disinfecting common areas at hospitals (such as rest rooms, halls, reception, corridors and lifts) |  |  |  |
| Cleaning and disinfecting surfaces and objects that are frequently touched, such as handles, elevator buttons, handrails, doorknobs and dispensers |  |  |  |
| Keeping the room where disinfectant solution is prepared used aerated and Labelling the prepared solution of disinfectant |  |  |  |
| Following the manufacturer’s instructions to ensure that disinfectants are prepared and handled safely. |  |  |  |
| Wearing appropriate PPE when visiting COVID-19 patient room |  |  |  |
| Donning and doffing PPEs appropriately |  |  |  |
| Following regular training on IPC including PPEs donning and doffing |  |  |  |
| I wash my hands before and after wearing PPEs |  |  |  |
| Handling laundry carefully to mitigate the risk of potential transmission. |  |  |  |
| Putting textiles, linens, and clothes in special, marked laundry bags |  |  |  |
| Washing laundry in warm cycles (60-90ºC) with the usual detergents. |  |  |  |
| Placing disposable items (hand towels, gloves, medical masks, tissues) in a container with a lid and following hospital action plan and national regulations for waste management. |  |  |  |
| Following COVID-29 news |  |  |  |
